# Subtype-Specific Prognosis, Recurrence Patterns, and Molecular Features in 148 Chinese Uterine Sarcomas: A Real-World Study

**DOI:** 10.3390/cancers18111689

**Published:** 2026-05-22

**Authors:** Ting Huang, Xinyu Xie, Xinqiao Du, Xiuling Sun, Guo Zhang, Jianliu Wang

**Affiliations:** 1 Department of Obstetrics and Gynecology, Peking University People’s Hospital, Beijing 100044, China; huangting@pkuph.edu.cn (T.H.); xzx5141@psu.edu (X.X.); 2411210340@stu.pku.edu.cn (X.D.); wangjianliu@pkuph.edu.cn (J.W.); 2 Department of Statistics, Eberly College of Science, The Pennsylvania State University, State College, PA 16802, USA

**Keywords:** uterine sarcoma, prognostic determinant, molecular characteristic, individualized therapy

## Abstract

Background: Uterine sarcomas are rare, heterogeneous malignancies with distinct pathological behaviors. This study aimed to identify clinicopathological characteristics, prognostic risk factors, and potential therapeutic targets to enhance clinical management. Methods: A retrospective analysis was conducted on 148 patients with uterine sarcoma treated at Peking University People’s Hospital between 1996 and 2025. Clinical outcomes, pathological subtypes, and immunohistochemical profiles were assessed. Additionally, bioinformatics analyses from RNA bulk sequencing of GEO datasets (GSE87581, GSE85383, GSE222045 and GSE64763) were performed to elucidate molecular characteristics across subtypes. Results: The most prevalent subtypes were uterine leiomyosarcoma (uLMS; 38.5%) and low-grade endometrial stromal sarcoma (LG-ESS; 29.7%). The 5-year recurrence rate was 50.5%, with frequent metastases to the pelvis and lungs. LG-ESS demonstrated the most favorable 5-year survival rate (90.3%), significantly higher than that of uLMS (61.8%) and undifferentiated uterine sarcoma (50.0%). Multivariate analysis identified histological subtype, stage, and coagulative necrosis as independent prognostic factors for overall and progression-free survival. Transcriptomic profiling revealed immunosuppression (*CSF1R/CSF3R* expression) in high-grade ESS, while uLMS exhibited activation of cell cycle and homologous recombination pathways. Conclusions: Histological subtype, stage, and coagulative necrosis were critical prognostic factors in uterine sarcoma. The findings suggest that vigilant pulmonary surveillance and further investigation into tailored therapeutic strategies may be warranted-including endocrine therapy for hormone-receptor-positive tumors, immunotherapy for high-grade ESS, and PARP inhibitors for uLMS. However, these hypotheses require thorough preclinical and clinical validation. Additionally, caution should be exercised to avoid overtreatment of chemotherapy in early-stage uLMS.

## Simple Summary

Uterine sarcomas are rare and aggressive uterine cancers. We studied 148 patients and public gene datasets to identify key prognostic factors and potential treatments. The most common subtypes were leiomyosarcoma and low-grade endometrial stromal sarcoma. Five-year survival was best for low-grade stromal sarcoma (90.3%) and worst for undifferentiated sarcoma (50.0%). Independent prognostic factors included subtype, stage, and coagulative necrosis. Gene analysis revealed immune suppression in high-grade stromal sarcoma and cell-cycle activation in leiomyosarcoma. Our results suggest that lung surveillance is important, and that tailored therapies—such as endocrine therapy, immunotherapy, or PARP inhibitors—may be worth investigating, but overtreatment with chemotherapy in early-stage leiomyosarcoma should be avoided.

## 1. Introduction

Uterine sarcomas represent a rare subset of gynecologic malignancies, constituting approximately 3% to 7% of all uterine cancers globally [1]. They are characterized by high aggressiveness and poor prognosis. The main pathological types of uterine sarcoma include uterine leiomyosarcoma (uLMS), low grade and high grade endometrial stromal sarcoma (LG-ESS and HG-ESS), undifferentiated uterine sarcoma (UUS), and adenosarcoma (AS) [2]. In China, uterine sarcomas account for 1.46% to 7% of uterine malignancies. According to available Chinese data, uLMS constitutes 29.8% to 44% of cases, LG-ESS accounts for 28% to 43.9%, HG-ESS comprises 11.4% to 16%, and AS represents 7% to 8%, with UUS marking up the remainder [3,4]. These regional statistics underscore the significant prevalence of uLMS and ESS within the Chinese population.

Differentiating uterine sarcomas from benign leiomyomas presents a substantial clinical challenge. The overlapping clinical and imaging characteristics often preclude a definitive preoperative diagnosis, necessitating reliance on histopathological evolution of the surgical specimen for final diagnosis [5]. In terms of surgical management, the necessity of lymph node dissection and adnexectomy in uterine sarcoma remains a topic of ongoing debate. Current evidence indicates that systematic lymphadenectomy is generally not recommended for most subtypes in the absence of evident nodal involvement. Conversely, bilateral salpingo-oophorectomy is often considered, particularly for hormone-receptor-positive tumors such as low-grade endometrial stromal sarcoma [6,7].

Several major clinical and research challenges persist. Firstly, there is considerable prognostic variability among histological subtypes, with LG-ESS demonstrating significantly longer survival than HG-ESS and uLMS [8]. However, the underlying mechanisms remain poorly understood; Secondly, treatment options are limited, and conventional modalities, including surgery, radiotherapy, and chemotherapy, yield suboptimal outcomes [9]. Thirdly, the low incidence of uterine sarcoma poses a barrier to the clinical investigation of novel therapies such as targeted agents and immunotherapies [10].

To address these gaps, we collected and analyzed clinicopathological data and follow-up information from patients with uterine sarcoma who were treated at Peking University People’s Hospital between 1996 and 2025. Our objective was to identify clinical features and prognostic risk factors. Additionally, to explore the molecular basis underlying subtype-specific behaviors, we employed bioinformatics analyses using RNA bulk sequencing data obtained from the GEO database (GSE222045, GSE85383, and GSE87581) to identify differentially expressed genes between HG-ESS and LG-ESS, as well as between uLMS and leiomyoma (LM). Our findings revealed that HG-ESS is characterized by immunosuppression, while uLMS exhibits enhanced proliferative capacity and homologous recombination deficiency compared to LM. These molecular distinctions have the potential to inform individualized therapeutic strategies for uterine sarcomas, though further validation is needed.

## 2. Materials and Methods

### 2.1. Patients and Data Collection

**Figure 1:**
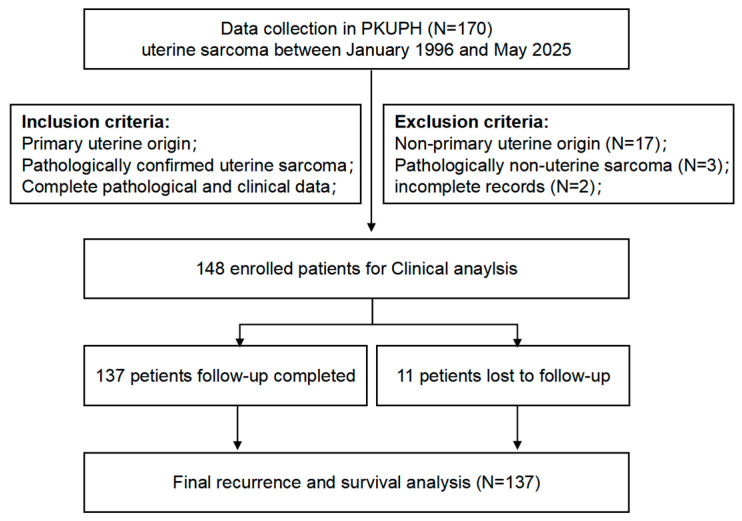
Flowchart of the study design.

Patients with uterine sarcoma treated at Peking University People’s Hospital from January 1996 to April 2025 were retrospectively reviewed. The inclusion criteria comprised: (1) postoperative histopathological confirmation of uterine sarcoma; (2) primary uterine origin, excluding metastases from other sites; and (3) availability of complete pathological and clinical data. The exclusion criteria included: (1) metastatic uterine sarcoma; (2) incomplete pathological or clinical records; and (3) loss to follow up without any outcome data (these patients were excluded from survival analysis only). Figure 1 provides a flow diagram illustrating the patient selection process.

The clinical data collected encompassed: (1) clinical characteristics, including age, body mass index (BMI), presenting symptoms, medical history, reproductive history, family history, and surgical details (including oophorectomy); (2) pathological characteristics, such as pathological subtype, lymph node metastasis, mitotic count, coagulative necrosis and immunohistochemical markers (ER, PR, CD10, SMA, desmin, Ki 67); and (3) prognostic information, comprising survival status, recurrence, and follow-up duration (months). The follow-up started from the date of initial diagnosis and ended on May 8, 2025. Overall survival (OS) was defined as time from treatment initiation to death from any cause. Progression-free survival (PFS) was defined as time from treatment initiation to disease progression or death.

### 2.2. GEO Data Acquisition and Differential Gene Analysis

To explore the molecular mechanisms underlying subtype-specific behaviors, we analyzed three independent GEO datasets. The GEO database was searched using the keyword “uterine sarcoma,” and the gene datasets GSE87581 and GSE85383, which include HG-ESS and LG-ESS, as well as GSE222045 and GSE64763, which include uLMS and LM, were selected. Each dataset was processed separately as described below. The detailed characteristics of each dataset are summarized in Table 1.

For the ESS datasets (GSE87581 and GSE85383), differentially expressed genes (DEGs) between HG-ESS and LG-ESS were identified separately for each dataset using the Limma package 3.62.2 (|fold change| ≥ 1.5, adjusted *p* value < 0.05). The two DEG lists were then intersected to obtain common DEGs that were consistently differentially expressed across both datasets, thereby reducing false positives. For the uLMS vs. LM comparison, DEGs were identified from GSE222045 alone using the same threshold. These genes were subsequently subjected to KEGG and GO enrichment analyses, and further protein–protein interaction (PPI) analysis. The top 50 genes ranked by interaction scores were selected for Friends analysis.

### 2.3. Statistical Analysis

All statistical analyses were conducted using GraphPad Prism 8.0 (GraphPad Software, Boston, MA, USA) and R software (version 4.2; R Foundation for Statistical Computing, Vienna, Austria). A two-sided *p* value of <0.05 was deemed statistically significant. Continuous variables were initially assessed for normality using the Shapiro–Wilk test. Variables following a normal distribution were expressed as mean ± standard deviation (SD) and compared between two groups using the independent two-sample *t*-test. For non-normally distributed continuous variables, the Mann–Whitney U test (non-parametric) was employed. Categorical variables were analyzed using the chi-square test when the expected frequency in each cell was ≥5; otherwise, Fisher’s exact test was used. Fisher’s exact test is preferred for sparse tables to avoid inaccurate approximations. OS and PFS were conducted using the Kaplan–Meier method, and survival curves were compared using the log-rank test. Cox proportional hazards regression was performed to identify independent prognostic factors. Variables with *p* < 0.10 in univariate analysis were entered into the multivariate model. The proportional hazards assumption was verified using Schoenfeld residuals; no violation was detected (*p* > 0.05 for all covariates). Results were reported as hazard ratios (HR) with 95% confidence intervals (CI). GraphPad Prism 8.0 was utilized to generate figures.

## 3. Results

### 3.1. Clinicopathological Characteristics of Patients with Uterine Sarcoma

**Figure 2:**
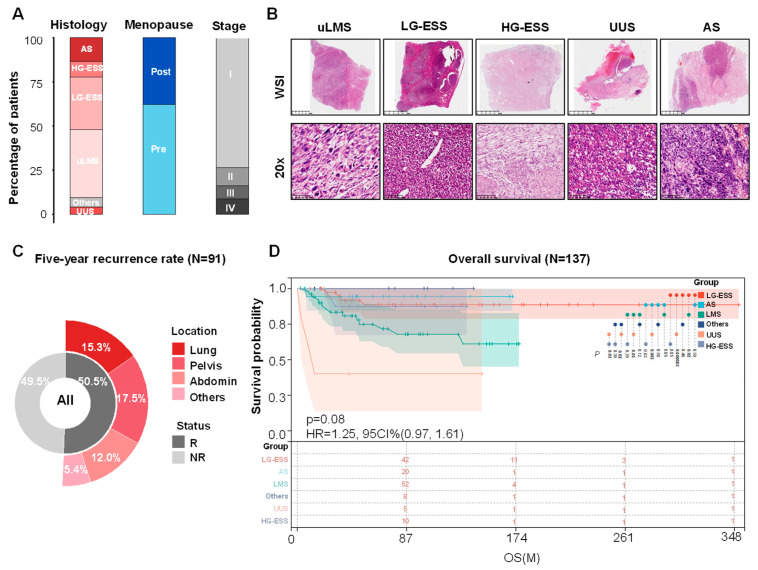
Clinicopathological characteristics and prognosis of uterine sarcoma. (**A**) Distribution of pathological types, menopausal status, and tumor stage in patients with uterine sarcoma; Different colors are used to represent different categories. (**B**) H&E staining of uterine sarcomas with different pathological types: up, whole slide image (scale bar, 5 mm); down, magnified view of the boxed area at 20× magnification (scale bar, 100 μm). (**C**) Pie chart showing 5-year survival rate and recurrence location of uterine sarcoma; (**D**) Overall survival of patients with different pathological types. Abbreviations: uLMS, uterine leiomyosarcoma; LG-ESS, low-grade endometrial stromal sarcoma; HG-ESS, high-grade endometrial stromal sarcoma; UUS, undifferentiated uterine sarcoma; AS, adenosarcoma; R, relapse; NR, Non-relapse.

The peak incidence of uterine sarcoma was between 40 and 60 years of age, the most common initial symptom was irregular vaginal bleeding (34.46%, 51/148), followed by abdominal pain, and rapid enlargement of a uterine fibroid detected during physical examination (Table 1). Fifty-six patients were postmenopausal at the time of diagnosis. Figure 2A illustrates the distribution of pathological types, menopausal status, and tumor stages among patients diagnosed with uterine sarcoma. The pathological types of the 148 uterine sarcomas included uLMS, LG-ESS, HG-ESS, UUS, AS, and others (Figure 2B). uLMS was identified as the predominant subtype, with 57 cases representing 38.51% of the total, followed by LG-ESS. Among the 148 patients, 80 underwent postoperative chemotherapy, while 9 received postoperative radiotherapy. Lymphadenectomy was performed in 37 patients, constituting 25% of the cohort. Additionally, oophorectomy was conducted in 117 patients. The rates of patients undergoing oophorectomy were 70.5% (31/44) for LG-ESS, 84.6% (11/13) for HG-ESS, and 83.3% (5/6) for UUS. In the case of uLMS, the proportion was 87.7% (50/57), as detailed in Table 2.

### 3.2. Prognostic Analysis of Uterine Sarcoma

Among the 148 patients enrolled, 11 were lost to follow-up, resulting in a follow-up rate of 92.6%. By the conclusion of the follow-up period, 42.3% (58/137) of the patients had experienced disease recurrence. Notably, among the 91 patients with a minimum follow-up of 5 years, the recurrence rate reached 50.5% (46/91). The median time to recurrence was calculated to be 19.3 months (min–max: 2.4–214.5 months). The pelvis and lungs were identified as the most prevalent sites for metastasis (Figure 2C). In terms of pathological types, the recurrence rate beyond 5 years post-diagnosis was 72.2% (26/36) for uLMS, with a median time to recurrence of 21.4 months (min–max: 3.3–179.7 months). For LG-ESS, the 5-year recurrence rate and median time to recurrence were 35.4% (11/31) and 47.7 months (min–max: 2.4–214.5 months), respectively. For HG-ESS, the 5-year recurrence rate and median time to recurrence were 66.6% (4/7) and 7.7 months (min–max: 3.7–9.7 months), respectively (Figure S1). Among the 91 patients with a disease duration of more than 5 years and complete follow-up data, only one received apatinib, 34 underwent postoperative chemotherapy based on anthracycline-containing regimens, 15 received other chemotherapy regimens, and the remaining 42 did not receive any chemotherapy. Further analysis of the relationship between recurrence and clinicopathological characteristics revealed that tumor type, karyokinesis, coagulative necrosis, and postoperative chemotherapy were significantly correlated with recurrence (*p* < 0.05) (Table 3).

A survival follow-up study was conducted on a cohort of 137 patients diagnosed with uterine sarcoma. The estimated OS rates at 5 years and 10 years were 83.2% (95% CI, 76.6–90.3%) and 80.7% (95% CI, 73.5–88.5%), respectively. Prognostic comparisons across different pathological subtypes indicated that patients with LG-ESS exhibited a significantly longer OS compared to those with uLMS (Figure 2D). The 5-year survival rate for uLMS patients was 61.8% (21/34), with stage-specific survival rates of 66.7% (16/24) for stage I, 100% (3/3) for stage II, 25% (1/4) for stage III, and 33.3% (1/3) for stage IV. In contrast, the 5-year survival rate for LG-ESS was 90.3% (28/31), with stage-specific rates of 92.3% (24/26) for stage I, 50% (1/2) for stage II, 100% (2/2) for stage III, and 0% (0/1) for stage IV. The 5-year survival rates for HG-ESS, UUS, and AS were 83.3% (5/6), 50.0% (1/2), and 100% (12/12), respectively.

**Figure 3:**
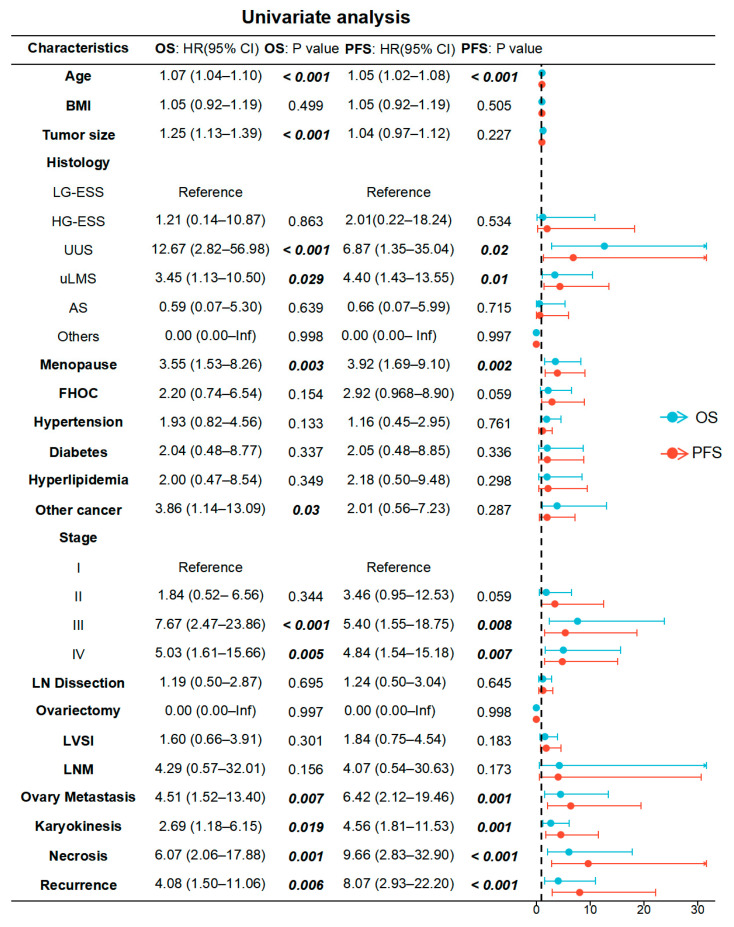
Univariate Cox analysis of prognosis in uterine sarcoma Abbreviations: BMI, body mass index; uLMS, uterine leiomyosarcoma; LG-ESS, low-grade endometrial stromal sarcoma; HG-ESS, high-grade endometrial stromal sarcoma; UUS, undifferentiated uterine sarcoma; AS, adenosarcoma; FHOC, family history of other cancers; LVSI, lymphovascular space invasion; LNM, lymph node metastasis; OS, overall survival; PFS, progression-free survival.

**Figure 4:**
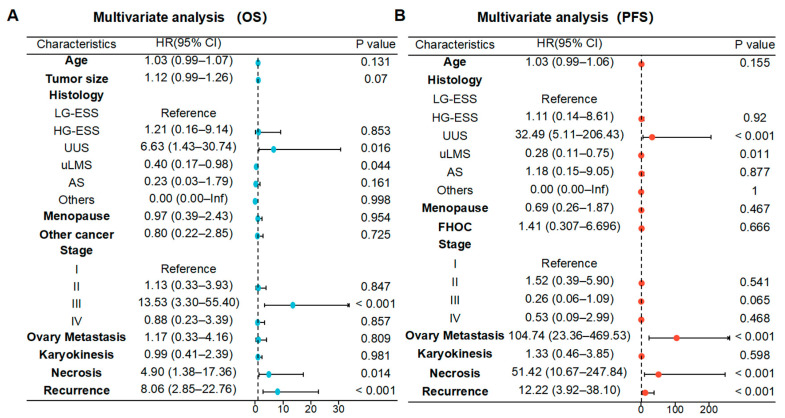
(**A**) Multivariate Cox analysis of prognosis in uterine sarcoma. Multivariate Cox analysis of PFS and uterine sarcoma; (**B**) Multivariate Cox analysis of OS and uterine sarcoma. Abbreviations: uLMS, uterine leiomyosarcoma; LG-ESS, low-grade endometrial stromal sarcoma; HG-ESS, high-grade endometrial stromal sarcoma; UUS, undifferentiated uterine sarcoma; AS, adenosarcoma; FHOC, family history of other cancers; OS, overall survival; PFS, progression-free survival.

Univariate Cox regression analysis identified that age, pathological subtype, menopausal status, tumor stage, ovarian metastasis, Karyokinesis (>10/10 HPF), coagulative necrosis, and chemotherapy were significantly associated with PFS. In contrast, factors such as age, tumor diameter, pathological type, menopausal status, history of other malignancies, tumor stage, ovarian metastasis, Karyokinesis (>10/10 HPF), coagulative necrosis, and postoperative chemotherapy were associated with OS in patients with uterine sarcoma. Ovarian metastasis and pelvic lymphadenectomy were not associated with either OS or PFS, and radiotherapy and endocrine therapy were also unrelated to patient prognosis (Figure 3). Further multivariate Cox analysis, incorporating factors with *p* < 0.1 from the univariate analysis, revealed that pathological type, mitotic figure, and coagulative necrosis were independent prognostic risk factors for both OS and PFS (Figure 4).

Further analysis of prognostic risk factors in patients with LG-ESS and uLMS using univariate Cox analysis revealed that, for uLMS, age (continuous variable), tumor diameter (continuous variable), stage (III–IV), lymph node metastasis, and ovarian metastasis (positive) were risk factors for OS. In contrast, menopause, family history of cancer, stage, lymph node metastasis, and ovarian metastasis were associated with PFS in uLMS. In summary, tumor stage, lymph node metastasis, and ovarian metastasis significantly influenced the prognosis of uLMS, including both OS and PFS (Figure S2a). In LG-ESS, increasing age was significantly associated with differences in both OS and PFS (Figure S2b). Neither pelvic lymphadenectomy nor oophorectomy had any impact on patient prognosis.

**Figure 5:**
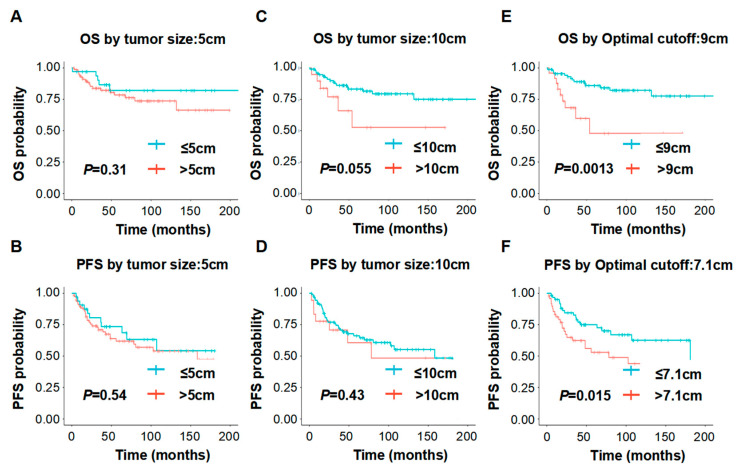
Exploration of the relationship between tumor diameter and prognosis in patients with uterine sarcoma. (**A**,**B**) Survival curves for OS and PFS with a tumor maximum diameter cutoff of 5 cm; (**C**,**D**) survival curves for OS and PFS with a tumor maximum diameter cutoff of 10 cm; (**E**) survival curve for OS at the tumor maximum diameter yielding the smallest *p*-value; (**F**) survival curve for PFS at the tumor maximum diameter yielding the smallest *p*-value; Abbreviations: OS, overall survival; PFS, progression-free survival.

Furthermore, we investigated whether tumor diameter was associated with the prognosis of uterine sarcoma. Analysis of the above results indicated that, when tumor diameter was treated as a continuous variable, larger tumor size was associated with worse OS. However, there is currently no standardized cutoff value for tumor diameter as a prognostic indicator. When the maximum tumor diameter in patients with uterine sarcoma was dichotomized at 5 cm or 10 cm, neither OS nor PFS showed a significant association with tumor diameter (*p* > 0.05) (Figure 5A–D). Further subtype analysis showed that findings for patients with uterine leiomyosarcoma were consistent with those for the overall cohort (Figure S3a–d), whereas PFS in LG-ESS was significantly associated with tumor diameter when a cutoff value of 5 cm was applied (Figure S4a–d). To determine the optimal tumor size cutoff for survival outcomes, we employed a data-driven approach using the minimum *p*-value method, a technique also validated in other malignancies [11,12,13] for identifying clinically relevant thresholds from continuous variables. We found that in the overall cohort, a diameter of 9 cm yielded a significant difference in OS between patients with tumors > 9 cm and those with tumors ≤ 9 cm (*p* = 0.0013), while a diameter of 7.1 cm yielded a significant difference in PFS between patients with tumors > 7.1 cm and those with tumors ≤ 7.1 cm (*p* = 0.015) (Figure 5E,F). In both uterine leiomyosarcoma and LG-ESS, the tumor diameter cutoff most strongly associated with OS was identical to that for the overall cohort (9 cm) (Figures S3e and S4e). For PFS, the tumor diameter cutoff most strongly associated with uLMS was 7.1 cm (Figure S3f), whereas that for LG-ESS was 3.5 cm (Figure S4f). In summary, the optimal tumor size cutoff for survival differs among pathological types of uterine sarcoma and warrants further investigation. These cutoffs were derived from a data-driven method and warrant validation in independent cohorts.

### 3.3. Association of ER/PR and Ki 67 with Subtypes and Prognosis of Uterine Sarcoma

**Figure 6:**
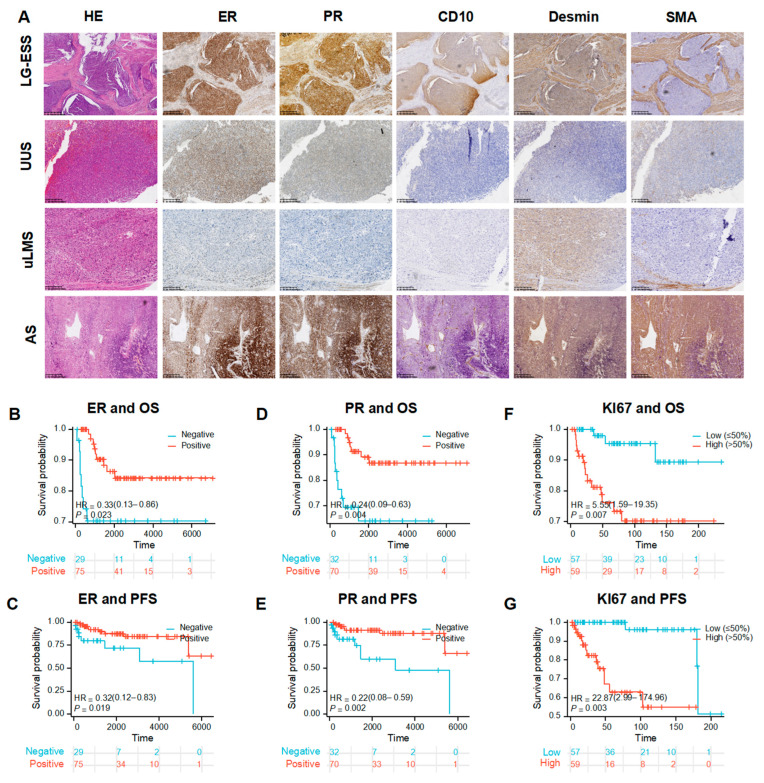
Pathological staining characteristics of uterine sarcoma and their association with histological subtypes and prognosis. (**A**) IHC images of different pathological subtypes of uterine sarcoma; Scale bar, 400 μm; (**B**) OS curve according to ER expression in patients with uterine sarcoma; (**C**) PFS curve according to ER expression in patients with uterine sarcoma; (**D**) OS curve according to PR expression in patients with uterine sarcoma; (**E**) PFS curve according to PR expression in patients with uterine sarcoma; (**F**) OS curve according to Ki 67 expression in patients with uterine sarcoma; (**G**) PFS curve according to Ki 67 expression in patients with uterine sarcoma; Abbreviations: uLMS, uterine leiomyosarcoma; LG-ESS, low-grade endometrial stromal sarcoma; UUS, undifferentiated uterine sarcoma; AS, adenosarcoma; OS, overall survival; PFS, progression-free survival; HE, hematoxylin and eosin; ER, estrogen receptor; PR, progesterone receptor. CD10, cluster of differentiation 10. SMA, smooth muscle actin.

Immunohistochemical markers, including estrogen receptor (ER), progesterone receptor (PR), and the proliferation index Ki 67, play important roles in the differential diagnosis and prognostic stratification of uterine sarcoma (Figure 6A). Positive expression of ER and PR is directly relevant to the selection of postoperative endocrine therapy.

Among patients with uterine sarcoma, ER expression showed significant association with prognosis, including OS and PFS (Figure 6B,C), and patients in the PR-positive group had significantly better prognosis than those in the PR-negative group (Figure 6D,E). Patients were divided into low (0–50%) and high (50–100%) Ki 67 expression groups. High Ki 67 expression was associated with significantly worse prognosis: the HR for OS was 5.55 (95% CI [1.59–19.35]), and the HR for PFS was 22.87 (95% CI [2.99–174.96]), suggesting that Ki67 ≥ 50% is a strong indicator of poor prognosis in patients with uterine sarcoma (Figure 6F,G).

Further analysis of ER/PR and Ki 67 expression by pathological subtype revealed that, among patients with LG-ESS, only 2 patients were ER-negative and 1 patient was PR-negative, indicating uniformly high ER and PR expression in LG-ESS, consistent with previous literature reports. In uterine leiomyosarcoma (uLMS), 48% of cases expressed ER and 43.4% expressed PR. Among all subtypes, UUS exhibited the highest proportion of Ki67 positivity, followed by uLMS, whereas LG-ESS showed the lowest proportion (Figure S5a). However, in uLMS, neither ER nor PR expression was significantly associated with OS or PFS (all *p* > 0.05). Furthermore, when patients with uLMS were stratified using the same 50% cutoff (0–50% vs. 50–100%), Ki67 expression was not significantly associated with either OS or PFS (*p* > 0.05 for both comparisons; Figure S5b–g).

### 3.4. Molecular Characterization of Uterine Sarcoma

To explore the molecular basis underlying the clinical differences observed above, particularly the aggressive nature of HG-ESS and uLMS, we next analyzed three independent GEO datasets. Two datasets (GSE87581, GSE85383) were used to compare HG-ESS vs. LG-ESS, and two dataset (GSE222045, GSE64763) to compare uLMS vs. LM. The differential expression and functional enrichment results are described below.

### 3.5. Gene Expression Profile of HG-ESS

**Figure 7:**
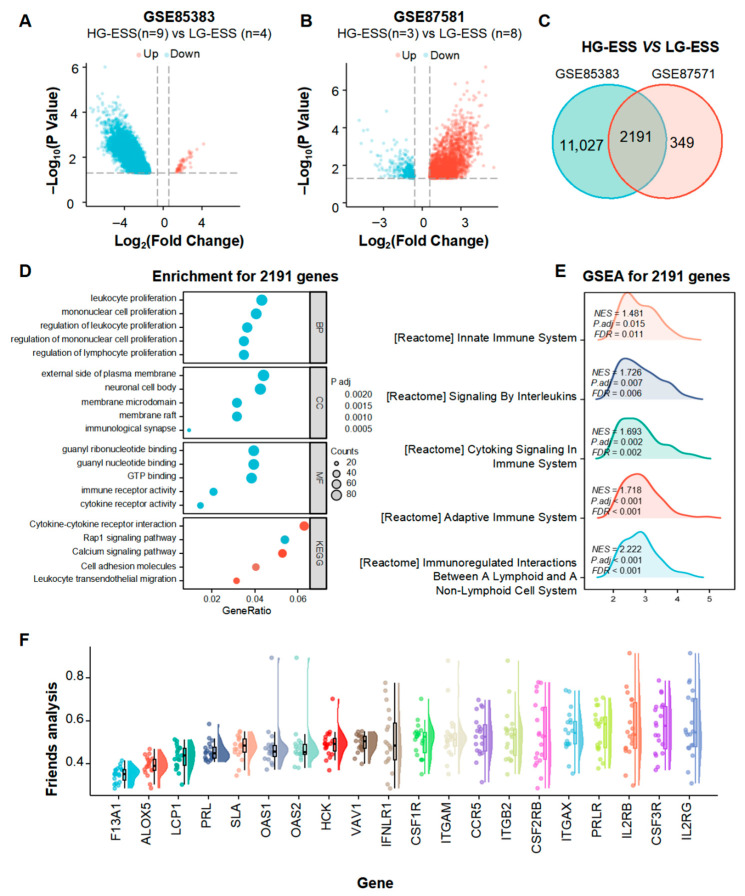
Molecular differences between LG-ESS and HG-ESS. (**A**) Volcano plot of DEGs between HG-ESS and LG-ESS in the GSE85383 dataset; (**B**) Volcano plot of DEGs between HG-ESS and LG-ESS in the GSE87581 dataset; (**C**) Venn diagram of DEGs shared between the two datasets; (**D**) Bubble plot of enrichment analysis for the 2191 shared DEGs; (**E**) GSEA ridge plot of the 2191 shared DEGs; (**F**) Friends analysis identifying hub genes in HG-ESS compared with LG-ESS. Abbreviations: LG-ESS, low-grade endometrial stromal sarcoma; HG-ESS, high-grade endometrial stromal sarcoma.

At the transcriptomic level, RNA sequencing data of HG-ESS and LG-ESS were collected and analyzed for differentially expressed genes (DEGs). Using an absolute fold change (FC) threshold of 1.5 and *p* < 0.05 as the screening criteria, a total of 13,218 and 2540 DEGs were identified in the GSE85383 and GSE87581 datasets, respectively, with 2191 DEGs shared between the two datasets (Figure 7A–C). Enrichment analysis of these 2191 differentially expressed genes revealed that the main enriched pathways were related to the proliferation of immune cells (granulocytes, monocytes, and lymphocytes) and chemokine signaling pathways (Figure 7D,E). Further PPI network analysis of genes enriched in immune-related pathways showed high expression of the neutrophil marker *CSF3R* and the macrophage marker *CSF1R*, suggesting an increased presence of tumor-associated macrophages and myeloid-derived suppressor cells (MDSCs) in HG-ESS. Several other DEGs were also notable: *CCR5* has been reported to recruit Treg cells and MDSCs, thereby suppressing antitumor immunity; *SLA* is a negative regulator of T-cell signaling that can inhibit T-cell activation; and *F13A1* promotes stromal fibrosis in tumors, which may impede immune cell infiltration (Figure 7F). Collectively, these findings indicate that the immune microenvironment of HG-ESS is predominantly characterized by myeloid cell–mediated immunosuppression, which may contribute to the aggressive clinical course and poorer survival of HG-ESS compared with LG-ESS observed in our clinical cohort.

### 3.6. Gene Expression Profile of uLMS

**Figure 8:**
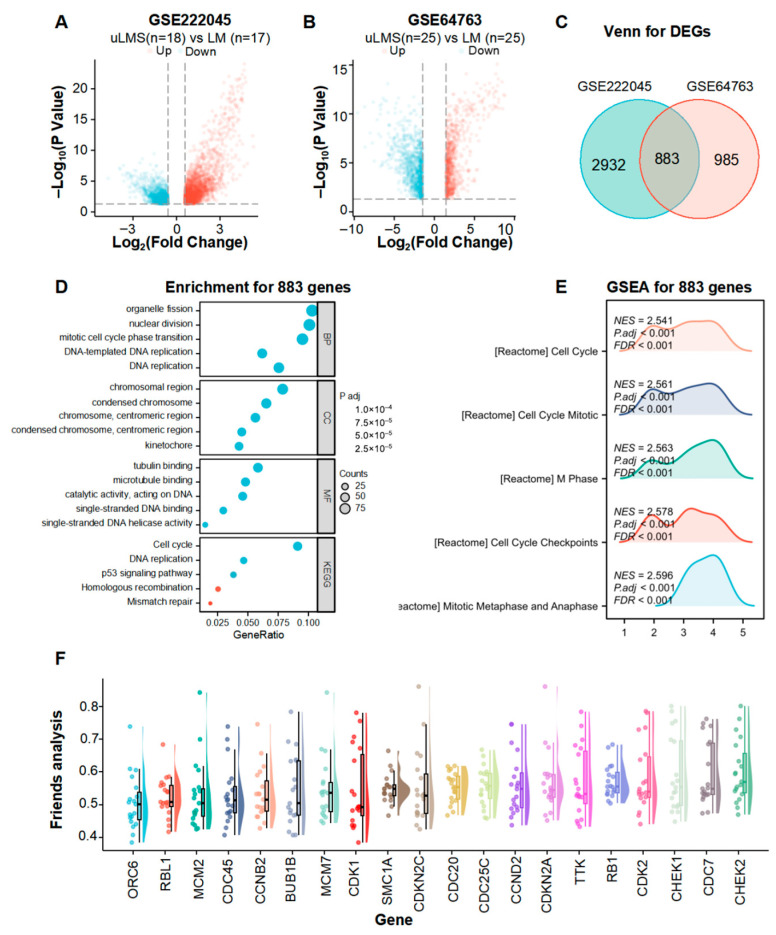
Molecular characteristic differences between uLMS and LM. (**A**) Volcano plot of DEGs between uLMS and LM in the GSE222045 dataset; (**B**) Volcano plot of DEGs between uterine uLMS and LM in the GSE64763 dataset; (**C**) Venn diagram of DEGs between the two datasets; (**D**) Bubble plot of enrichment analysis for the shared DEGs; (**E**) GSEA ridge plot of the 883 shared DEGs; (**F**) Friends analysis identifying hub genes in uLMS and LM. Abbreviations: uLMS, uterine leiomyosarcoma; LM, leiomyoma; DEG, differentially expressed genes.

Given the poor prognosis of uLMS shown in our clinical analysis, we further explored its molecular features. To elucidate the molecular characteristics distinguishing uLMS from LM, we retrieved RNA sequencing data for both tumor types from the GEO database and performed differential expression analysis. Using an absolute fold-change (|FC|) threshold of 1.5 and *p* < 0.05 as the selection criteria, we identified 3275 DEGs in the GSE222045 dataset and 1868 DEGs in the GSE64763 dataset, with 883 overlapping genes shared by both datasets (Figure 8A–C). Functional enrichment analysis of these 883 DEGs revealed predominant enrichment in pathways related to cell proliferation (including cell cycle, DNA replication, and mitosis), the p53 signaling pathway, and the homologous recombination deficiency (HRD) pathway (Figure 8D,E). Further PPI network analysis of genes enriched in cell proliferation–related pathways identified the top 20 hub genes, among which *CHEK1* and *CHEK2* regulate DNA damage checkpoints, contributing to increased genomic instability; *CDC20* and *CDC25C* control mitotic progression, promoting chromosomal instability; and *CDK1* and *CDK2* have been reported to drive cell cycle transitions, thereby facilitating cell proliferation (Figure 8F). Yoshida K et al. [14] reported that *CHEK1/2* inhibitors (Prexasertib) can significantly suppress the proliferation of uterine leiomyosarcoma cells. Prexasertib has already been approved by the FDA as a monotherapy for patients with platinum-resistant ovarian cancer and endometrial cancer. Our finding of *CHEK1/2* upregulation in uLMS thus suggests a potential therapeutic opportunity for this aggressive subtype.

## 4. Discussion

Uterine sarcomas are rare but highly heterogeneous, with marked differences in pathological characteristics and prognosis among subtypes. Surgery combined with adjuvant radiotherapy and chemotherapy is currently the most widely used treatment approach; however, challenges remain, including overtreatment with chemotherapy, unclear indications for endocrine therapy, and insufficient application of targeted agents. In this study, we summarized the clinical and prognostic data of 137 Chinese patients with uterine sarcoma, analyzed high-risk factors for recurrence and poor outcomes, and further examined the transcriptomic features of high-prevalence subtypes with unfavorable prognosis. These findings aim to provide guidance for clinical management and the rational application of novel targeted therapies in uterine sarcoma.

### 4.1. Prognostic Differences Between Uterine Sarcoma Pathological Types

uLMS and LG-ESS are the most common subtypes of uterine sarcoma, accounting for 38.5% and 29.7% of cases in this study, respectively, which is generally consistent with previous reports [15]. In this study, the Kaplan–Meier of the 137 patients with follow-up data estimated 5-year and 10-year OS rates were 83.2% (95% CI, 76.6–90.3%) and 80.7% (95% CI, 73.5–88.5%), respectively. These rates are higher than those reported by Sandra E [16] in a cohort of over 5000 cases (55% and 40%, respectively) and also exceed the 3-year OS rate of 60.2% reported by Dan L [4] for 114 patients from western China. This discrepancy may be attributed to the higher proportion of patients diagnosed at FIGO stage I in our cohort (73.6% vs. 54% and 71.9%, respectively). Patients with LG-ESS had a relatively favorable prognosis, with a 5-year OS rate of 90.63% (29/32), significantly higher than that of uLMS at 65.8% and HG-ESS at 83.3% (5/6). The 5-year recurrence rate for LG-ESS was 35.4%, markedly lower than 72.2% for uLMS. LG-ESS predominantly exhibited locoregional recurrence (pelvic and abdominal cavity), with a median time to recurrence (TTR) of 35 months, whereas uLMS most commonly recurred in the lung, with a median TTR of 21.4 months. This difference may be related to their distinct histologic origins, as uLMS more frequently undergoes hematogenous metastasis. Therefore, pulmonary monitoring should be strengthened for patients with uterine sarcoma, particularly those with uLMS.

### 4.2. Prognostic Risk Factor Analysis of Uterine Sarcoma

Most of the literature reports that pathological type, stage, and mitotic figure are prognostic risk factors for uterine sarcoma [17,18]. In our study, age, pathological type, menopausal status, tumor stage, ovarian metastasis, mitotic index, coagulative necrosis, and chemotherapy were common risk factors for both OS and PFS in univariate analysis. In multivariate analysis, histological subtype, mitotic index, and coagulative necrosis were independent prognostic factors for both OS and PFS, which is consistent with previous reports [19,20]. However, the relationship between chemotherapy and prognosis in uterine sarcoma remains controversial. Saqi B et al. found that chemotherapy could slightly prolong PFS (13.5 months vs. 11.0 months), with a small but statistically significant difference (*p* = 0.001) [21]. Brandon LS et al. [22] reported that chemotherapy did not improve survival in patients with early-stage uLMS but could benefit those with advanced disease (HR = 1.66, *p* < 0.001). In a phase III clinical trial (GOG-027) comparing gemcitabine plus docetaxel followed by doxorubicin versus observation in patients with stage I uLMS, chemotherapy reduced the risk of recurrence compared with observation (HR = 0.71), but no significant differences were observed in overall survival (chemotherapy group: 34.3 months vs. observation group: 46.4 months) or progression-free survival (chemotherapy group: 18.1 months vs. observation group: 14.6 months) [23]. Therefore, adjuvant chemotherapy is not recommended for patients with stage I uterine sarcoma. In our cohort of 148 patients, 80 received adjuvant chemotherapy postoperatively, including 51 (63.7%) with stage I disease, 12 (15%) with stage II, 9 (11.2%) with stage III, and 8 (10%) with stage IV. Chemotherapy was associated with an increased risk of recurrence (*p* < 0.001) and was an independent adverse prognostic factor for overall survival (HR = 5.97, *p* = 0.005). This may be attributable to overtreatment and substantial treatment-related toxicity. Current research is exploring combinations of chemotherapy with immunotherapy or gene therapy to enhance efficacy while reducing adverse effects. Literature reports indicate that local injection of human TP53 combined with chemotherapy significantly prolongs the interval to second recurrence in patients with uterine sarcoma [24], and chemotherapy combined with immunotherapy has shown superior outcomes compared with monotherapy in soft tissue sarcomas, including leiomyosarcoma [25]. Therefore, we should avoid overtreatment with chemotherapy in uterine sarcoma, and drug-sensitivity testing using patient-derived organoid cultures holds promise for guiding optimal chemotherapeutic selection.

### 4.3. Defining the Scope of Endocrine Therapy in Uterine Sarcoma

Consistent with previous reports [26], ER and PR were most highly expressed in LG-ESS. Among 27 patients who received endocrine therapy, 20 (74.1%) had LG-ESS and 3 (11.1%) had uLMS. In this study, 48% of uLMS cases expressed ER and 43.4% expressed PR. However, neither ER nor PR expression was significantly associated with PFS in uLMS. This contrasts with the findings of Mario ML et al. [27], who analyzed ER and PR expression in 43 uLMS cases and reported positivity rates of 43% and 41%, respectively, and noted that ER/PR-positive patients had markedly better PFS compared with ER/PR-negative patients. In a phase II clinical trial including only nine patients with early-stage uLMS, the oral letrozole group achieved 12-month and 24-month progression-free rates of 100%, whereas the observation group had rates of 80% and 40%, respectively [28]. The discrepancy may reflect differences in sample size or patient selection. Nevertheless, given our negative findings, the role of endocrine therapy in uLMS requires further investigation.

### 4.4. Exploration of Novel Therapeutic Approaches in Uterine Sarcoma

HG-ESS exhibits a shorter time to recurrence and worse prognosis compared with LG-ESS. The differentially expressed genes between the two subtypes are mainly enriched in pathways related to immune cell (granulocyte, monocyte, and lymphocyte) proliferation and chemokine signaling. HG-ESS demonstrates high expression of the neutrophil marker *CSF3R* and the macrophage marker *CSF1R*, suggesting an increased presence of tumor-associated macrophages (TAMs) and myeloid-derived suppressor cells (MDSCs). This indicates that HG-ESS may possess an immunosuppressive tumor microenvironment, a finding consistent with the study by Song et al. [29]. HG-ESS is characterized by significant infiltration of M2-polarized macrophages (*CD163*^+^, *CD206*^+^) and marked upregulation of T-cell exhaustion markers (*PD-1*, *CTLA-4*, *LAG-3*), implying potential immune evasion that contributes to poor outcomes. Therefore, strategies to overcome the immunosuppressive microenvironment hold promise for improving HG-ESS management. Notably, the *CSF1R* inhibitor pexidartinib, in combination with a PD-1 inhibitor, has been reported for the treatment of multiple solid tumors [30,31,32] and may represent a viable therapeutic option for HG-ESS.

uLMS is characterized by marked overexpression of cell cycle–related genes such as *CHEK1*, *CHEK2*, *CDC20*, and *CDK1*, accompanied by reduced *ESR1* and *PGR* expression, as well as loss of *BRCA1* and *BRCA2*. Enrichment analysis revealed that, compared with uterine leiomyoma, the differentially expressed genes in uLMS not only activate cell proliferation pathways but also stimulate homologous recombination repair and mismatch repair, indicating a molecular profile defined by overexpression of proliferation-related genes, concomitant homologous recombination deficiency (HRD), and reduced estrogen/progesterone responsiveness.

Accordingly, inhibitors targeting cell cycle regulatory genes hold promise for suppressing uLMS proliferation. The *CHEK1/2* inhibitor Prexasertib has demonstrated potent anti-uLMS effects in vitro and in animal models [14]. Furthermore, Nathan et al. [33] analyzed gene mutation profiles in 1236 soft tissue sarcomas and found that HR pathway–related gene mutations occurred in uLMS at twice the frequency observed in other soft tissue sarcomas, reaching 19%. Four patients with uLMS harboring *BRCA2* loss achieved durable clinical benefit from *PARP* inhibitors. Similarly, Chudasama et al. [34] reported *BRCA2* loss in 50% of uLMS cases, of which 40% were heterozygous losses and 10% were homozygous losses. These findings suggest that *PARP* inhibitors may represent a promising therapeutic option for HRD-positive uLMS; however, this strategy warrants further validation in large-scale clinical trials.

### 4.5. Limitations

Several limitations of this study should be acknowledged. First, this was a retrospective, single-center study with a relatively small sample size for certain histological subtypes (e.g., HG-ESS, *N* = 6; UUS, *N* = 2), which limits the statistical power of subgroup analyses and may introduce selection bias. Second, while we performed bioinformatics analyses to explore molecular mechanisms, we did not validate these findings in our patient cohort due to the poor quality and limited quantity of RNA extracted from archival formalin-fixed paraffin-embedded tissues. Thirdly, FIGO staging was based on the 2009 version for all patients. Although the 2023 revision was released during the study period, our use of only the main stage categories (I–IV)—which are largely unchanged between versions—means that no patient would be reclassified under the newer system. Finally, the follow-up duration varied (median 68.6 months, range 2.6–351.3 months), and 11 patients (7.4%) were lost to follow-up, which may introduce attrition bias. Despite these limitations, our study provides valuable real-world data on the clinical characteristics and prognosis of uterine sarcoma in a large Chinese cohort, and the bioinformatics findings offer testable hypotheses for future targeted therapy research.

## 5. Conclusions

**Figure 9:**
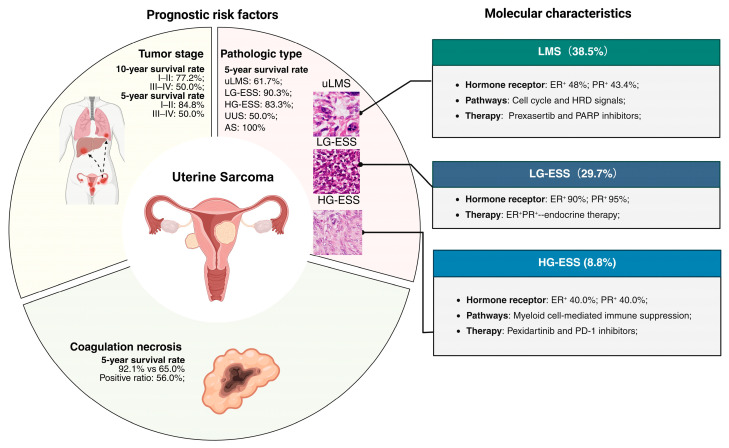
Schematic diagram of clinical characteristic of uterine sarcoma. Abbreviations: uLMS, uterine leiomyosarcoma; LG-ESS, low-grade endometrial stromal sarcoma; HG-ESS, high-grade endometrial stromal sarcoma; UUS, undifferentiated uterine sarcoma; AS, adenosarcoma; ER, estrogen receptor; PR, progesterone receptor; HRD, homologous recombination deficiency; PARP, poly (ADP-ribose) polymerase; PD-1, programmed cell death protein 1.

Uterine sarcomas are characterized by a low incidence, a high recurrence rate, and significant prognostic heterogeneity. Independent prognostic risk factors for both OS and PFS include pathological type, mitotic figures, and coagulative necrosis. LG-ESS demonstrated a favorable prognosis, with local recurrence, particularly in the pelvic and abdominal cavity, being most prevalent. In contrast, pulmonary recurrence was most frequently observed in uLMS, underscoring the necessity for enhanced lung monitoring during follow-up. In early-stage uLMS, the potential for overtreatment with chemotherapy should be considered; endocrine therapy may be discussed for patients with ER/PR-positive tumors, although our findings indicate that its benefits remain uncertain. HG-ESS exhibited an immunosuppressive phenotype, suggesting a potential advantage from immunotherapies such as anti-PD-L1 treatment, whereas the activation of HRD pathways in uLMS supports the potential application of PARP inhibitors (Figure 9). Through the analysis of clinical data from 148 patients with uterine sarcoma, this study identifies key prognostic determinants, providing an important basis for early screening of high-risk patients for postoperative recurrence and informing individualized follow-up strategies. The molecular findings offer preliminary insights for future translational research, but should not be interpreted as immediate clinical recommendations.

## Figures and Tables

**Table 1:** Detailed information of three GEO datasets.

Dataset	Platform	Sample Type	Comparison
GSE87581	Illumina HiSeq 2000	HG-ESS (*N* = 3), LG-ESS (*N* = 8)	HG-ESS vs. LG-ESS
GSE85383	Agilent-028004	HG-ESS (*N* = 4), LG-ESS (*N* = 9)	HG-ESS vs. LG-ESS
GSE222045	Illumina NovaSeq 6000	uLMS (*N* = 18), LM (*N* = 17)	uLMS vs. LM
GSE64763	Affymetrix U133A 2.0	uLMS (*N* = 25), LM (*N* = 25)	uLMS vs. LM

**Table 2:** Clinical characteristics of 148 patients with uterine sarcoma.

Characteristics	*N* = 148	%
***Demographic***		
**Age**	49 ± 12.80	
**BMI**	23.76 ± 3.57 kg/m^2^	
**Menopause**		
No	92	62.2
Yes	56	37.8
**Births**		
0	15	10.1
1	93	62.8
2	31	20.9
≥3	9	6.1
**Previous cancer history**		
No	142	95.9
Yes	6	4.1
***Clinical presentation***		
**Abormal bleeding**		
No	97	65.5
Yes	51	34.5
**Rapid growth of myoma**		
No	138	93.2
Yes	10	6.8
**Abdominal pain**		
No	136	91.9
Yes	12	8.1
***Comorbidities***		
**Hypertension**		
No	111	75
Yes	37	25
**Diabetes**		
No	137	92.6
Yes	11	7.4
**Hyperlipidermia**		
No	142	95.9
Yes	6	4.1
***Pathology***		
**Stage**		
I	109	73.6
II	15	10.1
III	11	7.4
IV	13	8.8
**Histology**		
uLMS	57	38.5
LG-ESS	44	29.7
HG-ESS	13	8.8
UUS	6	4.1
AS	20	13.5
Others	8	5.4
***Treatment***		
**Neoadjuvant therapy**		
No	145	98.0
Yes	3	2.0
**Chemotherapy**		
No	68	45.9
Yes	80	54.1
**Radiation**		
No	139	93.9
Yes	9	6.1

**Table 3:** Analysis of risk factors for recurrence with onset more than 5 years. Italicized text indicates categories. Bold text indicates the content within each category.

Characteristics	R	NR	*p*	Statistic
***N***	**46**	**45**		
***Demographic***				
**Age, mean ± sd**	47.957 ± 13.281	47.644 ± 13.432	0.91	0.11
**Menopause, *N* (%)**			0.71	0.14
No	30 (33%)	31 (34.1%)		
Yes	16 (17.6%)	14 (15.4%)		
**Family history, *N* (%)**			0.28	1.16
No	40 (44%)	43 (47.3%)		
Yes	6 (6.6%)	2 (2.2%)		
**Previous cancer history, *N* (%)**			1.00	0.00
No	44 (48.4%)	43 (47.3%)		
Yes	2 (2.2%)	2 (2.2%)		
***Surgical procedure***				
**Surgical approach, *N* (%)**			0.48	0.51
Transabdominal	37 (43.5%)	32 (37.6%)		
Laparoscopic	7 (8.2%)	9 (10.6%)		
**lymphadenectomy, *N* (%)**			0.38	0.78
No	25 (31.6%)	23 (29.1%)		
Yes	13 (16.5%)	18 (22.8%)		
**Ovariectomy, *N* (%)**			0.14	2.14
Yes	33 (39.8%)	37 (44.6%)		
No	9 (10.8%)	4 (4.8%)		
***Pathology***				
**Histology** **, *N* (%)**			***0.00***	18.80
uLMS	26 (28.6%)	8 (8.8%)		
LG-ESS	11 (12.1%)	20 (22%)		
Others	2 (2.2%)	4 (4.4%)		
AS	2 (2.2%)	10 (11%)		
HG-ESS	4 (4.4%)	2 (2.2%)		
UUS	1 (1.1%)	1 (1.1%)		
**Stage, *N* (%)**			0.24	4.17
I	32 (35.2%)	39 (42.9%)		
III	5 (5.5%)	2 (2.2%)		
IV	3 (3.3%)	2 (2.2%)		
II	6 (6.6%)	2 (2.2%)		
**Tumor size, median (IQR)**	7.05 (5.45, 8.7)	6.7 (5, 8.4)	0.54	
**LVSI, *N* (%)**			0.55	0.36
No	39 (42.9%)	36 (39.6%)		
Yes	7 (7.7%)	9 (9.9%)		
**LNM, *N* (%)**			1.00	0.00
No	45 (49.5%)	44 (48.4%)		
Yes	1 (1.1%)	1 (1.1%)		
**Ovary metastasis, *N* (%)**			0.37	0.80
No	42 (46.2%)	44 (48.4%)		
Yes	4 (4.4%)	1 (1.1%)		
**Karyokinesis, *N* (%)**			***0.00***	15.53
≤10	22 (24.2%)	39 (42.9%)		
>10	24 (26.4%)	6 (6.6%)		
**Necrosis, *N* (%)**			***0.00***	10.80
Yes	28 (30.8%)	12 (13.2%)		
No	18 (19.8%)	33 (36.3%)		
***Adjuvant therapy***				
**Chemotherapy, *N* (%)**			***0.00***	11.99
No	13 (14.3%)	29 (31.9%)		
N-based	23 (25.3%)	11 (12.1%)		
Others	10 (11%)	5 (5.5%)		
**Radiotherapy, *N* (%)**			0.22	1.54
No	41 (45.1%)	44 (48.4%)		
Yes	5 (5.5%)	1 (1.1%)		
**Endocrine therapy, *N* (%)**			0.16	1.95
No	40 (44%)	34 (37.4%)		
Yes	6 (6.6%)	11 (12.1%)		

## Data Availability

All data are available from the corresponding author upon reasonable request.

## Supplementary Materials

The following supporting information can be downloaded at: https://www.mdpi.com/article/10.3390/cancers18111689/s1, Figure S1: Five-year recurrence rate and sites of recurrence. (a) Pie chart showing 5-year survival rate and recurrence location of LMS; (b) Pie chart showing 5-year survival rate and recurrence location of LG-ESS; Figure S2: Subgroup analysis of prognostic factors in uterine sarcoma; Figure S3: Association between tumor diameter and prognosis of LG-ESS. (a,b) Survival curves for OS and PFS with a tumor maximum diameter cutoff of 5 cm; (c,d) survival curves for OS and PFS with a tumor maximum diameter cutoff of 10 cm; (e) survival curve for OS at the tumor maximum diameter yielding the smallest *p*-value; (f) survival curve for PFS at the tumor maximum diameter yielding the smallest *p*-value; Figure S4: Association between tumor diameter and prognosis of uLMS. (a,b) Survival curves for OS and PFS with a tumor maximum diameter cutoff of 5 cm; (c,d) survival curves for OS and PFS with a tumor maximum diameter cutoff of 10 cm; (e) survival curve for OS at the tumor maximum diameter yielding the smallest *p*-value; (f) survival curve for PFS at the tumor maximum diameter yielding the smallest *p*-value; Figure S5. Differences in Ki67 expression among pathological types of uterine sarcoma and its association with prognosis. (a) Differences in Ki67 expression among different pathological types of uterine sarcoma; (b) OS curve according to ER expression in uLMS patients; (c) PFS curve according to ER expression in uLMS patients; (d) OS curve according to PR expression in uLMS patients; (e) PFS curve according to R expression in uLMS patients; (f) OS curve according to Ki 67 expression in uLMS patients; (g) PFS curve according to Ki 67 expression in uLMS patients; *** *p* < 0.001.

## Abbreviations

uLMS	uterine leiomyosarcoma
ESS	endometrial stromal sarcoma
LG-ESS	low-gradeendometrial stromal sarcoma
HG-ESS	high-gradeendometrial stromal sarcoma
UUS	undifferentiated uterine sarcoma
AS	adenosarcoma
LM	leiomyoma
OS	Overall survival
PFS	Progression-free survival
PPI	protein–protein interaction
SD	standard deviation
HR	hazard ratios
CI	confidence intervals
MDSCs	myeloid-derived suppressor cells
FC	fold change
DEGs	differentially expressed genes
HRD	Homologous recombination deficiency
TTR	time to recurrence
TAMs	tumor-associated macrophages
PARP	poly (ADP-ribose) polymerase
