# Supplementary material for: Subtype-Specific Prognosis, Recurrence Patterns, and Molecular Features in 148 Chinese Uterine Sarcomas: A Real-World Study
Source: Cancers (Basel). 2026 May 22;18(11):1689. doi: 10.3390/cancers18111689 (PMC13255855; doi:10.3390/cancers18111689)
Supplement: Supplementary file 1 [file cancers-18-01689-s001.zip › cancers-4319280-supplementary.pdf]

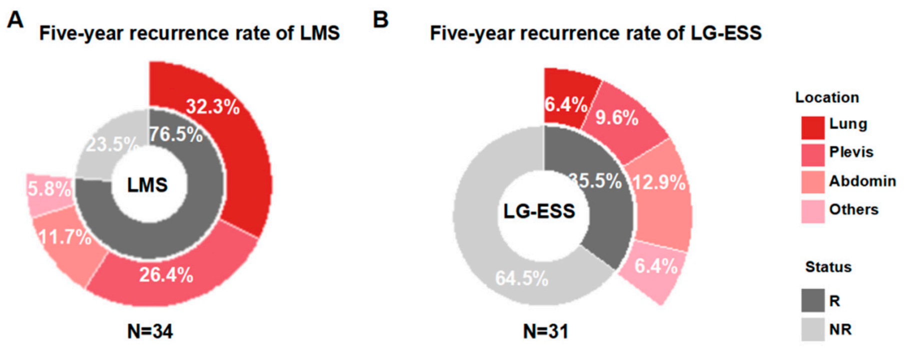

Figure S1 Five-year recurrence rate and sites of recurrence

(a) Pie chart showing 5-year survival rate and recurrence location of LMS; (b) Pie chart showing 5-year survival rate and recurrence location of LG-ESS.

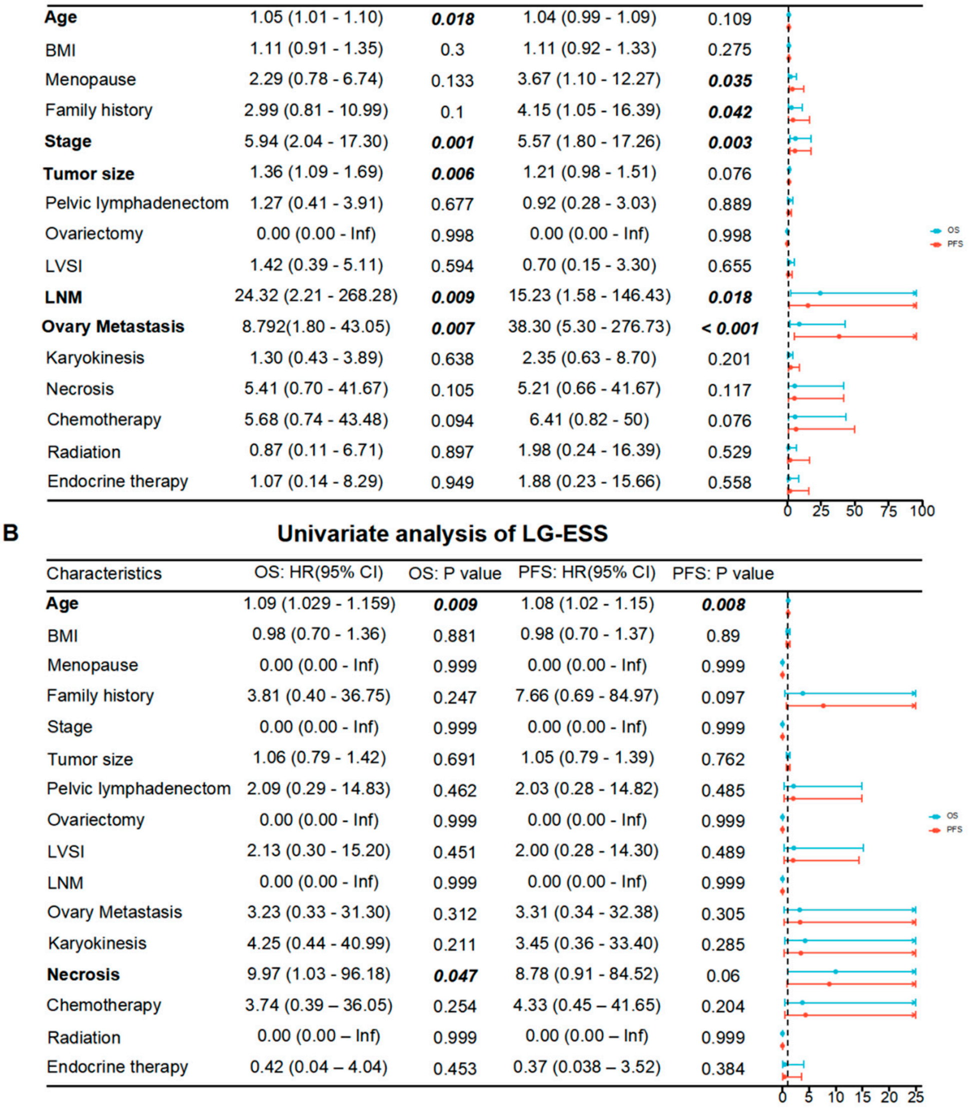

A

Univariate analysis of LMS

Characteristics	OS: HR(95% CI)	OS: P value	PFS: HR(95% CI)	PFS: P value
**Age**	1.05 (1.01 - 1.10)	***0.018***	1.04 (0.99 - 1.09)	0.109
BMI	1.11 (0.91 - 1.35)	0.3	1.11 (0.92 - 1.33)	0.275
Menopause	2.29 (0.78 - 6.74)	0.133	3.67 (1.10 - 12.27)	***0.035***
Family history	2.99 (0.81 - 10.99)	0.1	4.15 (1.05 - 16.39)	***0.042***
**Stage**	5.94 (2.04 - 17.30)	***0.001***	5.57 (1.80 - 17.26)	***0.003***
**Tumor size**	1.36 (1.09 - 1.69)	***0.006***	1.21 (0.98 - 1.51)	0.076
Pelvic lymphadenectom	1.27 (0.41 - 3.91)	0.677	0.92 (0.28 - 3.03)	0.889
Ovariectomy	0.00 (0.00 - Inf)	0.998	0.00 (0.00 - Inf)	0.998
LVSI	1.42 (0.39 - 5.11)	0.594	0.70 (0.15 - 3.30)	0.655
**LNM**	24.32 (2.21 - 268.28)	***0.009***	15.23 (1.58 - 146.43)	***0.018***
**Ovary Metastasis**	8.792(1.80 - 43.05)	***0.007***	38.30 (5.30 - 276.73)	***< 0.001***
Karyokinesis	1.30 (0.43 - 3.89)	0.638	2.35 (0.63 - 8.70)	0.201
Necrosis	5.41 (0.70 - 41.67)	0.105	5.21 (0.66 - 41.67)	0.117
Chemotherapy	5.68 (0.74 - 43.48)	0.094	6.41 (0.82 - 50)	0.076
Radiation	0.87 (0.11 - 6.71)	0.897	1.98 (0.24 - 16.39)	0.529
Endocrine therapy	1.07 (0.14 - 8.29)	0.949	1.88 (0.23 - 15.66)	0.558

B

Univariate analysis of LG-ESS

Characteristics	OS: HR(95% CI)	OS: P value	PFS: HR(95% CI)	PFS: P value
**Age**	1.09 (1.029 - 1.159)	***0.009***	1.08 (1.02 - 1.15)	***0.008***
BMI	0.98 (0.70 - 1.36)	0.881	0.98 (0.70 - 1.37)	0.89
Menopause	0.00 (0.00 - Inf)	0.999	0.00 (0.00 - Inf)	0.999
Family history	3.81 (0.40 - 36.75)	0.247	7.66 (0.69 - 84.97)	0.097
Stage	0.00 (0.00 - Inf)	0.999	0.00 (0.00 - Inf)	0.999
Tumor size	1.06 (0.79 - 1.42)	0.691	1.05 (0.79 - 1.39)	0.762
Pelvic lymphadenectom	2.09 (0.29 - 14.83)	0.462	2.03 (0.28 - 14.82)	0.485
Ovariectomy	0.00 (0.00 - Inf)	0.999	0.00 (0.00 - Inf)	0.999
LVSI	2.13 (0.30 - 15.20)	0.451	2.00 (0.28 - 14.30)	0.489
LNM	0.00 (0.00 - Inf)	0.999	0.00 (0.00 - Inf)	0.999
Ovary Metastasis	3.23 (0.33 - 31.30)	0.312	3.31 (0.34 - 32.38)	0.305
Karyokinesis	4.25 (0.44 - 40.99)	0.211	3.45 (0.36 - 33.40)	0.285
**Necrosis**	9.97 (1.03 - 96.18)	***0.047***	8.78 (0.91 - 84.52)	0.06
Chemotherapy	3.74 (0.39 – 36.05)	0.254	4.33 (0.45 – 41.65)	0.204
Radiation	0.00 (0.00 – Inf)	0.999	0.00 (0.00 – Inf)	0.999
Endocrine therapy	0.42 (0.04 – 4.04)	0.453	0.37 (0.038 – 3.52)	0.384

Figure S2 Subgroup analysis of prognostic factors in uterine sarcoma

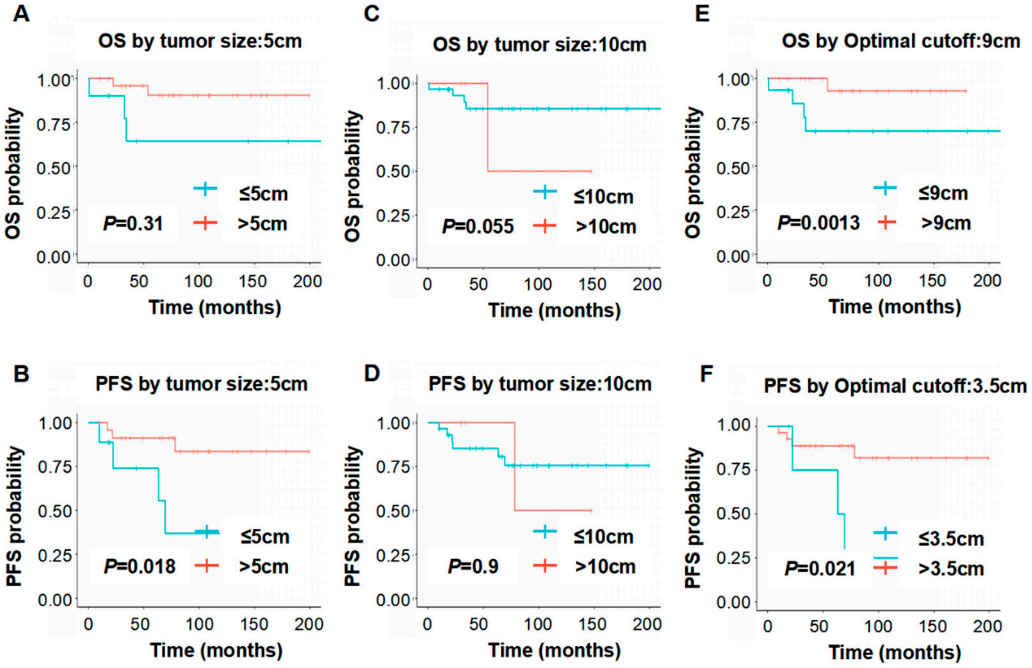

Figure S3 Association between tumor diameter and prognosis of LG-ESS
(a-b) Survival curves for OS and PFS with a tumor maximum diameter cutoff of 5 cm; (c-d) survival curves for OS and PFS with a tumor maximum diameter cutoff of 10 cm; (e) survival curve for OS at the tumor maximum diameter yielding the smallest P-value; (f) survival curve for PFS at the tumor maximum diameter yielding the smallest P-value;

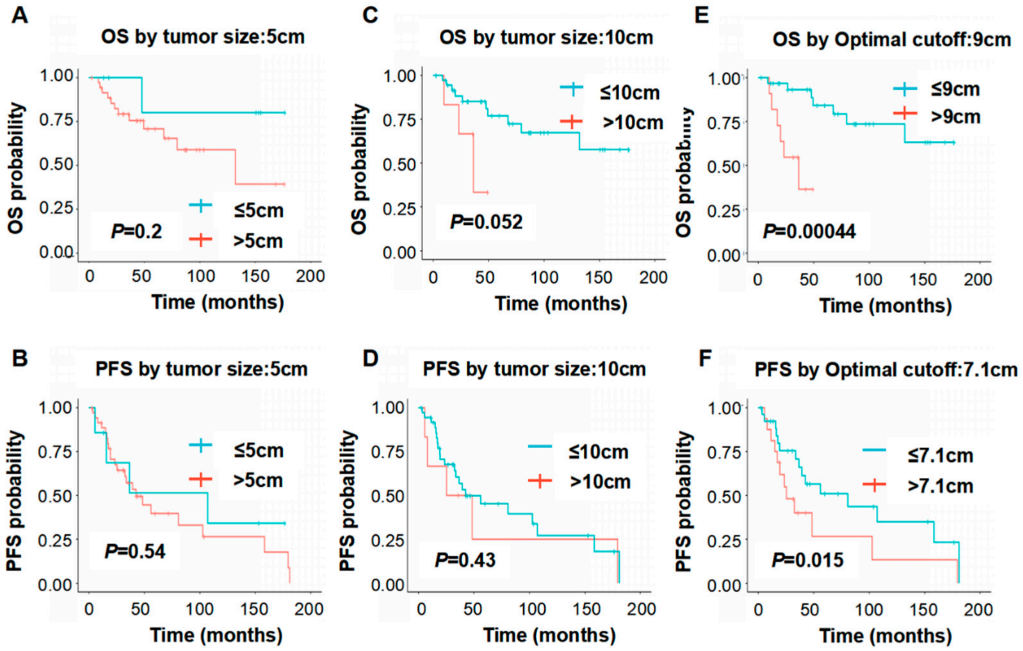

Figure S4 Association between tumor diameter and prognosis of uLMS
(a-b) Survival curves for OS and PFS with a tumor maximum diameter cutoff of 5 cm;

(c-d) survival curves for OS and PFS with a tumor maximum diameter cutoff of 10 cm; (e) survival curve for OS at the tumor maximum diameter yielding the smallest P-value; (f) survival curve for PFS at the tumor maximum diameter yielding the smallest P-value;

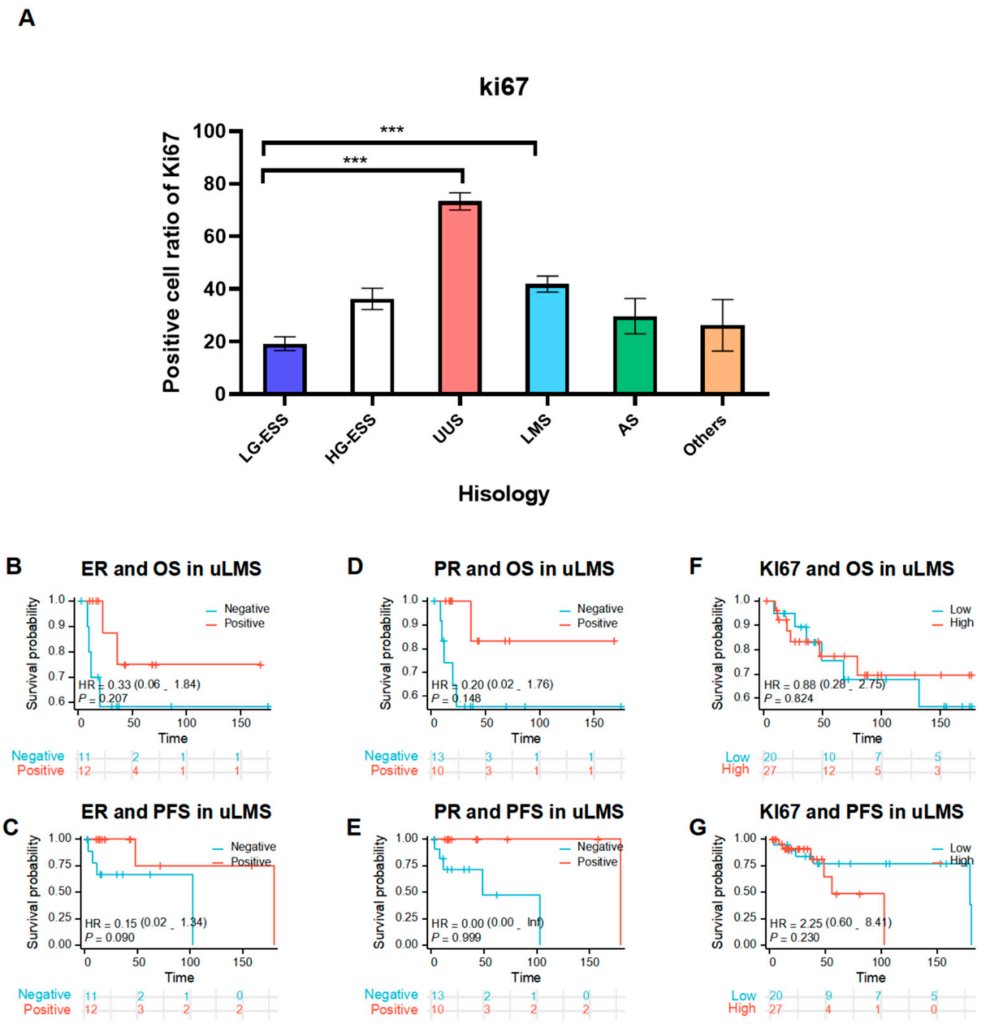

Figure S5 Differences in Ki67 expression among pathological types of uterine sarcoma and its association with prognosis

(a) Differences in Ki67 expression among different pathological types of uterine sarcoma; (b) OS curve according to ER expression in uLMS patients; (c) PFS curve according to ER expression in uLMS patients; (d) OS curve according to PR expression in uLMS patients; (e) PFS curve according to R expression in uLMS patients; (f) OS curve according to Ki 67 expression in uLMS patients; (g) PFS curve according to Ki 67 expression in uLMS patients; ***p<0.001.
